# Apoptosis Induced by Knockdown of uPAR and MMP-9 is Mediated by Inactivation of EGFR/STAT3 Signaling in Medulloblastoma

**DOI:** 10.1371/journal.pone.0044798

**Published:** 2012-09-12

**Authors:** Ramaprasada Rao Kotipatruni, Arun Kumar Nalla, Swapna Asuthkar, Christopher S. Gondi, Dzung H. Dinh, Jasti S. Rao

**Affiliations:** 1 Department of Cancer Biology and Pharmacology, University of Illinois College of Medicine at Peoria, Peoria, Illinois, United States of America; 2 Department of Neurosurgery, University of Illinois College of Medicine at Peoria, Peoria, Illinois, United States of America; University of Chicago, United States of America

## Abstract

**Background:**

Medulloblastoma is a highly invasive cancer of central nervous system diagnosed mainly in children. Matrix metalloproteinase-9 (MMP-9) and urokinase plasminogen activator receptor (uPAR) are over expressed in several cancers and well established for their roles in tumor progression. The present study is aimed to determine the consequences of targeting these molecules on medulloblastoma progression.

**Methodology/Principal Findings:**

Radiation is one of the foremost methods applied for treating cancer and considerable evidence showed that radiation elevated uPAR and MMP-9 expression in medulloblastoma cell. Therefore efforts are made to target these molecules in non-irradiated and irradiated medulloblastoma cells. Our results showed that siRNA-mediated knockdown of uPAR and MMP-9, either alone or in combination with radiation modulated a series of events leading to apoptosis. Down regulation of uPAR and MMP-9 inhibited the expression of anti-apoptotic molecules like Bcl-2, Bcl-xL, survivin, XIAP and cIAPI; activated BID cleavage, enhanced the expression of Bak and translocated cyctochrome C to cytosol. Capsase-3 and -9 activities were also increased in uPAR- and MMP-9-downregulated cells. The apoptosis induced by targeting MMP-9 and uPAR was initiated by inhibiting epidermal growth factor receptor (EGFR) mediated activation of STAT3 and NF-κB related signaling molecules. Silencing uPAR and MMP-9 inhibited DNA binding activity of STAT3 and also reduced the recruitment of STAT3 protein at the promoter region of Bcl-2 and survivin genes. Our results suggest that inhibiting uPAR and MMP-9 reduced the expression of anti-apoptotic molecules by inactivating the transcriptional activity of STAT3. In addition, treating pre-established medulloblastoma with siRNAs against uPAR and MMP-9 both alone or in combination with radiation suppressed uPAR, MMP-9, EGFR, STAT3 expression and induced Bak activation leading to apoptosis.

**Conclusion/Significance:**

Taken together, our results illustrated that RNAi mediated targeting of uPAR and MMP-9 might have therapeutic potential against medulloblastoma.

## Introduction

Medulloblastoma, the most common malignant brain tumor in childhood [1], are neuro-epithelial tumors arising from neural stem cell precursors in the granular cell layer of the cerebellum [2]. Despite the improved combination of surgery, radiation and chemotherapy, the outcome of medulloblastomas remains poor due to the difficulty in removing the highly invasive intracranial tumor radically and the short- and long-term adverse effects of conventional post-surgical adjuvant therapies [3]. Tumor cells acquire these invasive and metastatic characteristics mainly due to their ability to produce and activate proteolytic enzymes, such as serine, metallo- and cysteine proteases, which are able to degrade extracellular matrix (ECM) components and break down natural barriers, thereby aiding in tumor invasion and metastasis [4]. Urokinase plasminogen activator receptor (uPAR) plays a vital role in tumor invasion and progression by regulating proteolysis, activation of other matrix proteinases, growth factors and activates several intracellular signaling pathways [5], [6]. Matrix metalloproteinases (MMPs) play an important role in tissue repair, tumor invasion and metastasis [7]. The generation and analysis of transgenic and knockout mice for both MMPs and tissue inhibitors of MMPs have revealed that MMPs also play key roles in the process of carcinogenesis [8]. Radiotherapy, the most common mode of treating cancer, has been reported to elicit an activated phenotype that promotes rapid and persistent remodeling of the extracellular matrix (ECM) through the induction of proteases like MMP-9, uPA and uPAR [9].

Apoptosis is a programmed cell death involved in many physiological and pathological regulations [10]. Understanding of the mechanisms underlying apoptosis has resulted in the development of new strategies for treating illnesses and several clinical trials are under way. The apoptotic pathway consists of several triggers, modulators, and effectors. Signal transducers and activators of transcription (STAT) is constitutively expressed in high-grade gliomas activated by epidermal growth factor receptor (EGFR) [11]. The EGFR/STAT3 oncogenic pathway plays a central role in tumorigenesis by mediating cellular growth signals initiated by uPAR and α5β1 integrins [12]. One among the signalling pathway activated by EGFR is STAT proteins, which are reported to be elevated in a variety of solid tumors and hematologic malignancies [13]. STATs are known to have dual roles as a cytoplasmic signaling protein and nuclear transcription factor and activate a diverse set of genes, including some that are implicated in malignant progression [14]. STAT3 is found to be constitutively activated in medulloblastoma [15], and the level of STAT3 activation in medulloblastoma exceeds that of all other brain tumors examined, including glioblastoma, ependymomas, and astrocytomas [16].

Similar to STAT3, the NF-κB cascade has been reported to play an important role in the control of cell growth, differentiation, apoptosis, inflammation, stress response and many other physiologic processes involved in cellular signalling. NF-κB is a key protein and an important transcription factor that has been described as a major therapeutic target in cancer [17]. NF-κB can be activated by many types of stimuli, including TNF-α, UV radiation, free radicals, etc. The activity of NF-κB depends on its nuclear translocation capacity and binding to NF-κB-specific DNA binding sites, and in turn, regulation of transcription of genes involved in cell survival and apoptosis [18]. It has been reported that over expression of NF-κB protects cells from apoptosis, whereas inhibition or absence of NF-κB induces apoptosis or sensitizes cells to apoptosis-inducing agents, including ionizing radiation. [19].

RNA interference-based, targeted silencing of gene expression is a strategy of potential interest for cancer therapy [20]. Currently, attempts are being made to overcome the adverse effects and limitations of radiation-resistant tumor cells using a combination of gene therapy and radiotherapy. In the present study, to better characterize the key roles of uPAR and MMP-9 *in vitro* and *in vivo*, we have used a bicistronic plasmid vector expressing small hairpin RNAs (shRNAs) directed against both uPAR and MMP-9. Functional analyses revealed that the abrogation of both uPAR and MMP-9 expression inhibits proliferation and induces apoptotic cell death. These results indicate that the simultaneous knockdown of uPAR and MMP-9 using RNAi vectors is a promising tool for analysis of the function of downstream signaling pathways as well as the potential vectors for medulloblastoma cancer gene therapy in combination with radiation treatment.

## Results

### Plasmids Expressing shRNA Against uPAR and MMP-9 Effectively down Regulate the Target Genes in Medulloblastoma Cell Lines

**Figure 1:**
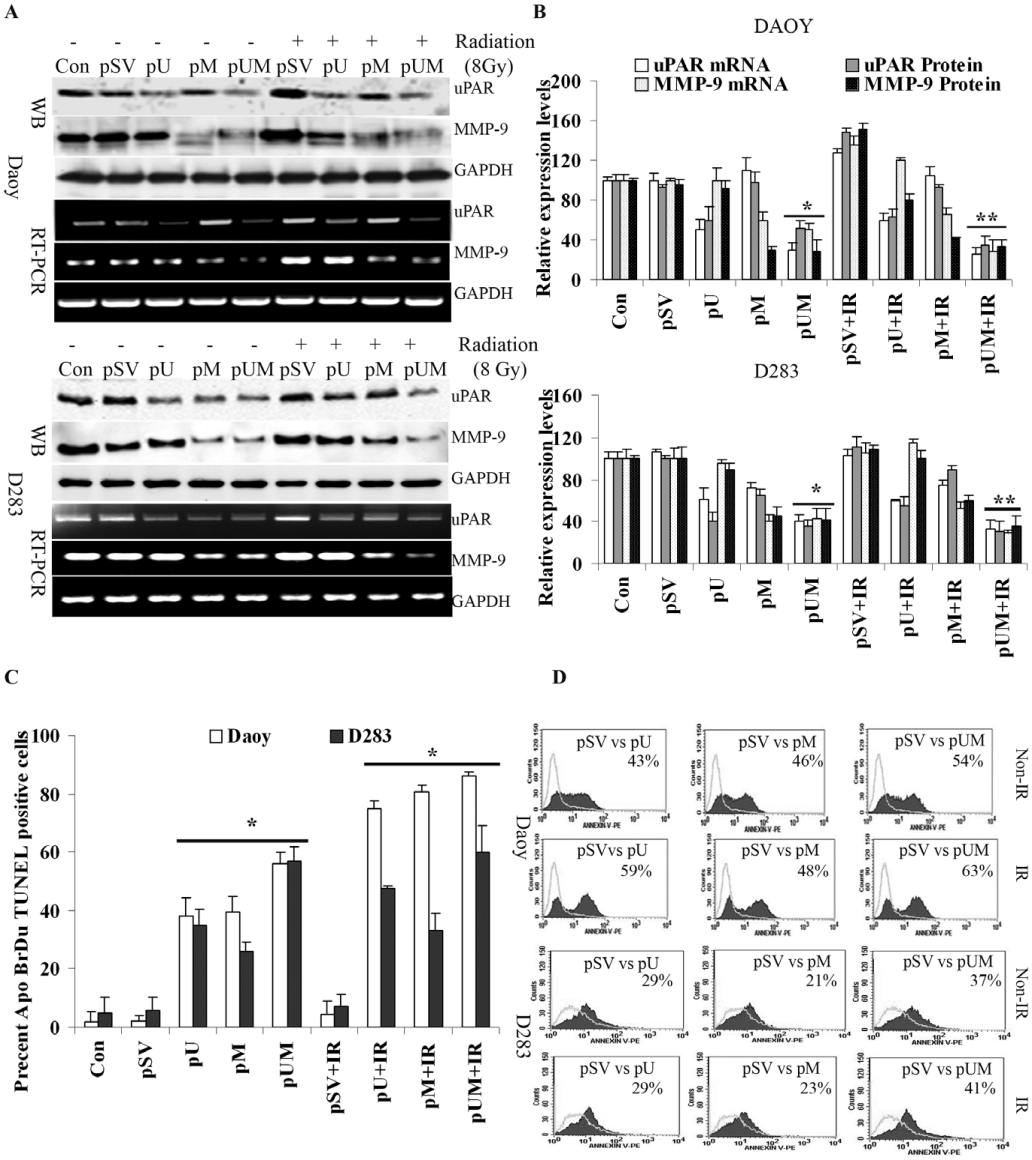
Transfection with pU, pM and pUM in combination with radiation specifically down regulates expression of uPAR and MMP-9 and induces apoptosis. Daoy and D283 cell lines were transfected with either transfection reagent alone (control, Con), pSV (scrambled vector) or gene specific shRNA’s as described in materials and methods. **A)** RT-PCR analysis and western blotting was carried to determine the expression levels of uPAR and MMP-9 in cell transfected with reagent, pSV, pU-, pM- and pUM-transfected Daoy and D283 cells (with and without radiation, 8 Gy). The experiments were repeated three times and representative images were shown. The immunoblots were stripped and re-probed with GAPDH as a loading control. Semi-quantitative RT-PCR analysis was carried out to detect mRNA levels of uPAR and MMP-9 using specific primers. **B)** Protein band and PCR amplicon intensities were quantified by densitometry analysis using ImageJ software (National Institutes of Health). The levels of uPAR and MMP-9 protein were normalized to GAPDH levels in mock-transfected cells. *Columns:* mean of triplicate experiments; *bars*: s.d.; *p<0.01, significant difference from pSV-transfected cells. **C)** 72 hrs after Transfection (with or without radiation), the cells were trypsinized and analyzed by flow cytometry to measure the number of apoptotic TUNEL-positive cells using the APO-BrdU TUNEL Assay kit. The percent apoptotic cells from each treatment are represented and the mean ± s.d. from three separate experiments was represented; *p<0.05 and ** *p*<0.01 were considered significant compared to pSV-transfected cells and pSV+IR treated cells, respectively. **D)** Apoptotic cells were also quantified using an Annexin V assay followed by FACS analysis. Results are reported as the percent of cells (minimum 10,000 analyzed) that were Annexin V-positive. Gating was based on positive and negative control cells. Representative FACS data of Daoy and D283 are shown (*n* = 3).

To study functional importance of uPAR and MMP-9 in medulloblastoma progression, Daoy and D283 cells were transfected with shRNA plasmids targeted against uPAR (pU), MMP-9 (pM), either alone or simultaneously (pUM) in combination with radiation (IR) treatment and compared with cells transfected with either transfection reagent (control/mock) or pSV (vector with scrambled non-specific sequence). Analyzing the mRNA levels isolated from the transfected cells with specific primers clearly showed the efficacy of these constructs in silencing the respective target gene. RT-PCR analysis demonstrated pUM transfection reduced both uPAR and MMP-9 transcripts levels by nearly ∼75% and 50%, respectively compared to the control and pSV-transfected in Daoy cells. While pUM treatment reduced uPAR and MMP-9 mRNA levels in D283 cells by nearly 60% and 59%, respectively (Figs. 1A and B). IR treatment alone augmented uPAR and MMP-9 transcript levels in Daoy cells by ∼30% and 50%, respectively. In D283 cells IR induced uPAR and MMP levels by only 10 and 25%, respectively. Similar observations were made when total cell lysates are immunoprobed with the specific antibodies. Cells treated with IR (pSV+IR) showed an increase in uPAR expression by 35% and 10% in Daoy and D283 cells, respectively compared with the respective non-irradiated cells (pSV alone). While IR elevated the expression of MMP-9 by ∼36% and 25% in Daoy and D283 cells, respectively. Transfecting medulloblastoma cells with pU, pM and pUM plasmids significantly inhibited uPAR and MMP-9 protein levels. uPAR and MMP-9 were reduced by nearly 55% and 60%, respectively in pUM-transfected Daoy cells compared to the cells transfected with pSV (Figs. 1A and 1B). While combination of pUM and IR treatment reduced the expression of uPAR and MMP-9 by nearly 65% and 75%, respectively as compared to the cells treated with pSV and IR. Similarly, D283 cells transfected with pUM showed that uPAR and MMP-9 protein levels were reduced by nearly 45% and 51%, respectively (Figs. 1A and 1B). In combination with radiation, pUM treatment significantly reduced both uPAR and MMP-9 levels by 59% and 65%, respectively compared to D283 cells treated with pSV and IR. The combination of siRNA and IR treatments not only inhibited the expression levels of the target genes, but inhibited the radiation induced expression of uPAR and MMP-9. Moreover, our results confirmed that bicistronic plasmid (pUM) was effective in targeting two molecules simultaneously than targeting a single gene by monocistromic plasmids (pU and pM).

### Inhibition of uPAR and MMP 9 Induces Apoptosis in Medulloblastoma Cells

Reduction in the cell number of pU, pM and pUM transfected cells made us to investigate the effect of uPAR and MMP-9 downregulation on medulloblastoma cells progression. Based on Apo-BrdU assay, a sub-lethal dosage (8 Gy) of IR has shown a non-significant slight increase in apoptotic cell percentage compared to the pSV transfected cells, but the percentage of apoptotic cells were significantly high in pU, pM and pUM transfected medulloblastoma cells. The percentage of apoptotic cell in pUM-transfected cell population was nearly 60% in Daoy cells and 62% in D283 cells compared to the respective pSV-transfected cells. Further, the combination of pUM and IR treatment increased the number of apoptotic cells by nearly 82% Daoy and was only 65% in D283 cell line, compared to combination of pSV and IR treatment (Fig. 1C). We further confirmed the apoptosis induced by uPAR and MMP-9 downregulation in medulloblastoma by Annexin V/PE staining assay. Overlaying the graphs (representing the number of Annexin V/PE positive cells) showed a shift in the number of Annexin V stained cells with pU, pM and pUM (with and without radiation) treatments compared to pSV-treated cells (Fig. 1D). The shift clearly indicates that pU, pM and pUM treatments significantly increased the number of annexin V-stained cells, which is an indication of apoptotic cells. Percent annexin V stained cells in each treatment is represent in Figure 1D.

### uPAR and MMP-9 shRNA Treated Induces BID Activation, Cytochrome c Release into the Cytosol and Loss of MMP (Ψm) in Medulloblastoma Cells

**Figure 2:**
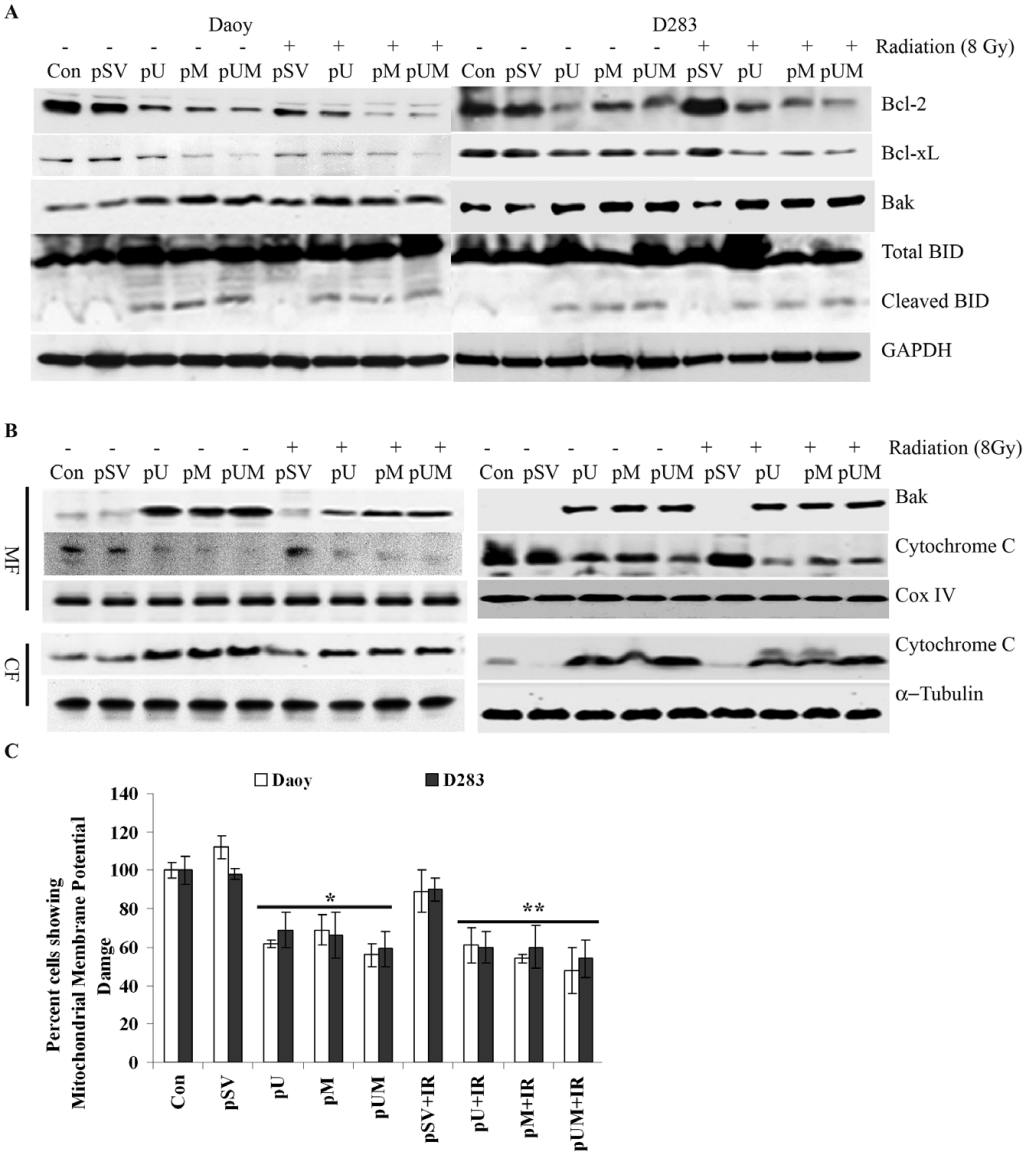
uPAR and MMP-9 gene silencing induced mitochondrial apoptosis critically depends on Bak activation and cytochrome C release into cytosol in Daoy and D283 cells. **A)** 72 hrs after transfecting Daoy and D283 cells with pU, pM and pUM (with or without radiation, 8 Gy), the cells were collected and analyzed by western blotting with the indicated antibodies. The membrane was reprobed with GAPDH to confirm equal protein loading. **B)** Sub-cellular fractions were prepared from the transfected cells as described in materials and methods. Cytochrome C release from mitochondria into the cytosol and mitochondrial Bak levels were determined by Western blotting of mitochondrial and cytosolic protein extracts. Membranes were re-probed with α-tubulin (cytosolic marker) or COX IV (mitochondrial marker). **C)** Mitochondrial membrane potential damage was evaluated using MitoLight green (Millipore). To determine the initiation of mitochondrial apoptosis in shRNA transfected Daoy and D283 cells, we carried out FACS analysis of mitochondrial membrane potential by sorting cells as described in materials and methods. Graphs represents percent number of cells showing reduced mitochondrial membrane potential in Daoy and D283 cell transfected with shuPAR, shMMP9 and shuPAR-MMP9 (treated with and without radiation). Each experiment is repeated 3 times * *p*<0.05 were statistically significant compared to pSV-transfected cells. A total of 10,000 cells were sorted per treatment.

To identify the molecular events underlying the induction of apoptosis in uPAR and MMP-9 knockdown medulloblastoma cells, we initially focused on expression pattern of Bcl-2 family members. Down regulation of uPAR and MMP-9 reduced the expression of anti-apoptotic molecules (Bcl-2 and Bcl-xL) and conversely the level of pro-apoptotic molecule, Bak was enhanced. Western blot analysis of total cell lysates extracted from shRNA-transfected cells (with and without radiation treatment) revealed the cleavage of BID to minor fragment (Fig. 2A). The functional significance of these expression patterns derives the involvement of cleaved BID (an 18 kDa protein) in activation of Bak. Further, western blot analysis of sub-cellular fractions showed that transfection with pU, pM and pUM (either alone or in combination with radiation) significantly increased cytosolic cytochrome C levels compared to pSV-transfected cells (Fig. 2B). Conversely, cytochrome C levels were reduced in the mitochondrial fraction of uPAR and MMP-9 downregulated cells. Apart from reduction of mitochondrial cytochrome C, a significant increase in mitochondrial Bak levels was also observed in pU-, pM- and pUM-transfected medulloblastoma cells compared to pSV-transfected cells (Fig. 2B). For normalization and to confirm equal loading of the above sub-cellular fractions, the blots were stripped and re-probed with COX IV (mitochondrial marker) and α-tubulin (cytosolic marker). The changes observed in cytosolic translocation of mitochondrial proteins caused us to determine the effect of down regulating uPAR and MMP-9 on mitochondrial membrane potential (*Ψm*). As expected, pUM transfection showed that nearly 49% of Daoy and 44% of D283 cells lost their mitochondrial membrane potential compared to control cells. Whereas IR and pUM treatment increased the percentage of cell which had has lost their mitochondrial membrane potential by 55% (Daoy) and 59% (D283) compared to cell treated with pSV and IR (Fig. 2C).

### Silencing uPAR and MMP-9 Initiates Caspase-9, Caspase-3 and PARP Cleavage

**Figure 3:**
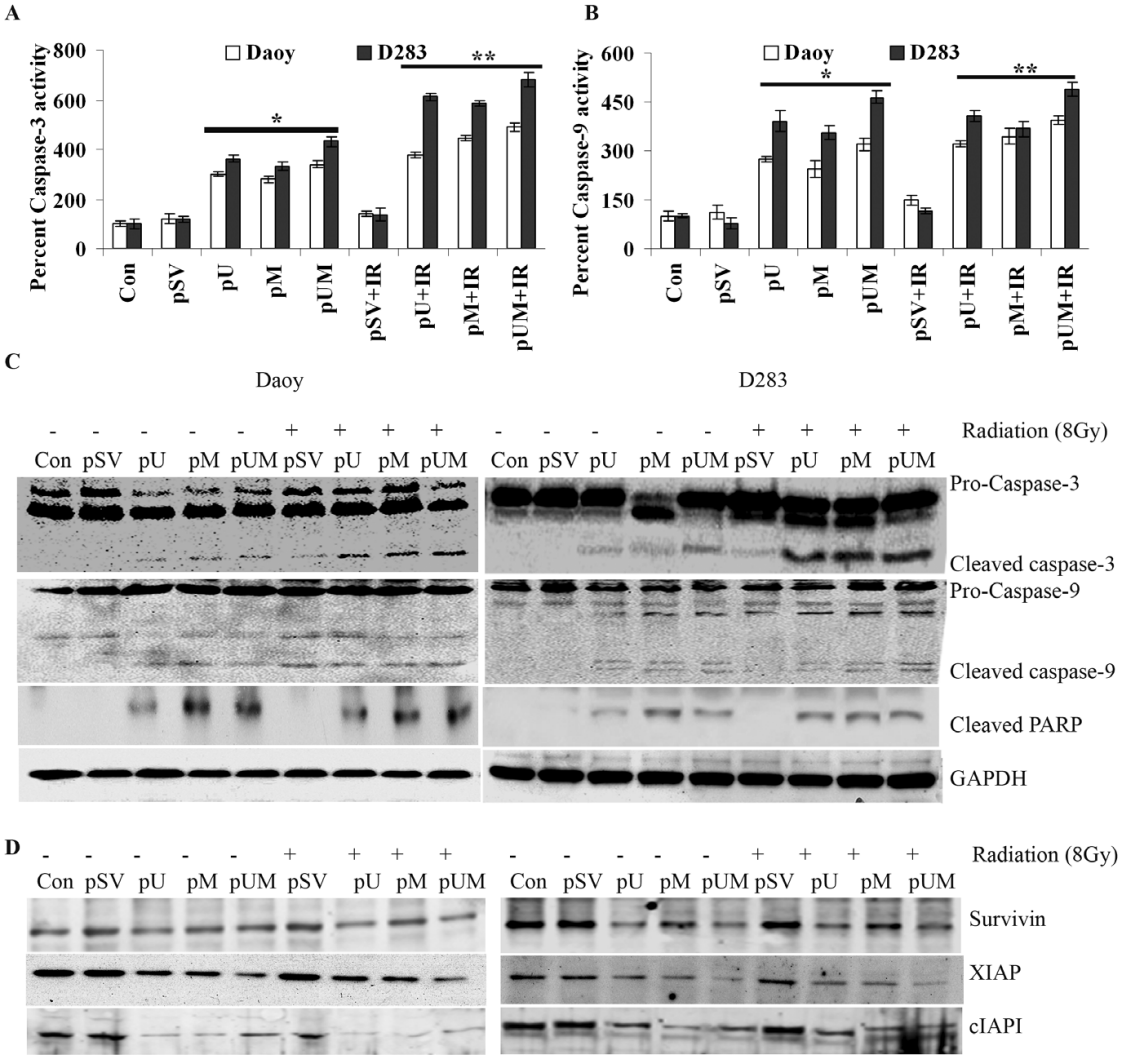
Transcriptional silencing of uPAR and MMP-9 activates caspase-3, caspase-9 and PARP. Activity of caspase-3 and caspase-9 in Daoy and D283 cells was determined using **A)** caspase-3 and **B)** caspase-9 colorimetric activity kits. Total cell lysates collected from transfected cells (with and with radiation) were collected, incubated with the respective peptide substrate, and treated with caspase conjugate p-nitroaniline (Ac-DEVD-pNA). Further, activity was measured at 405 nm using a microplate reader. Y-axis shows the percent activity of the respective caspase by normalizing the control to 100%. Bars represent the means ± s.d. from three independent experiments. * *p*<0.01 and ** *p<*0.05 were statistically significant compared to control and pSV-transfected cells. **C)** Immunoblot analysis of proteins isolated from cells treated with pU, pM and pUM (with and without radiation) was carried out to determine the activities of caspase-3, caspase-9 and PARP cleavage. **D)** The above immunoblots were stripped and re-probed with survivin, XIAP and cIAP1 specific antibodies, to determine the expression levels of inhibitory proteins.

Increased cytosolic cytochrome C and Bak:Bcl-2 ratio caused us to determine the effect of uPAR and MMP-9 down regulation in activation of caspases. To investigate this, we initially measured the activity of caspase-3 and caspase-9 in cells treated with pU, pM or pUM (with and without radiation). Based on the fluorescence units measured, we confirmed nearly 50-60% higher caspase-3 activity in pUM-treated cells (with and without IR) (Fig. 3A). Similarly, caspase-9 activity was significantly increased by ∼70–80% when cells were transfected with pUM (in combination with IR) in both Daoy and D283 cells (Fig. 3B). Since increased caspase activity is associated with cleavage of the caspase, we next immunoprobed the total cell lysates with specific antibodies to confirm the cleavage of capsaspes in uPAR and MMP-9 downregulated cells. Western blot analysis confirmed enhanced cleavage of the caspases-3 and -9 molecules in pUM transfected cells compared to either control or pSV treatment cells (Fig. 3C). Similarly, we found the cleavage of PARP 1, a 85 kDa cleaved fragment, was significantly higher in uPAR and MMP-9-downregulated cells as compared to the control and pSV-transfected cells (Fig. 3C). Re-probing immunoblots with anti-survinin, anti-XIAP and anti-cIAPI antibodies showed that the expression levels of these inhibitory apoptotic proteins were significantly inhibited by 30%, 69% and 50%, respectively following treatment of pUM plasmid in Daoy (Fig. 3D). Similarly, pUM treatment reduced the expression of survinin, XIAP and cIAPI in D283 cells by nearly 63, 57 and 51%, respectively compared to pSV treated cells.

### Blockade of uPAR and MMP9 Inhibited EGFR Mediated Activation of STAT3

**Figure 4:**
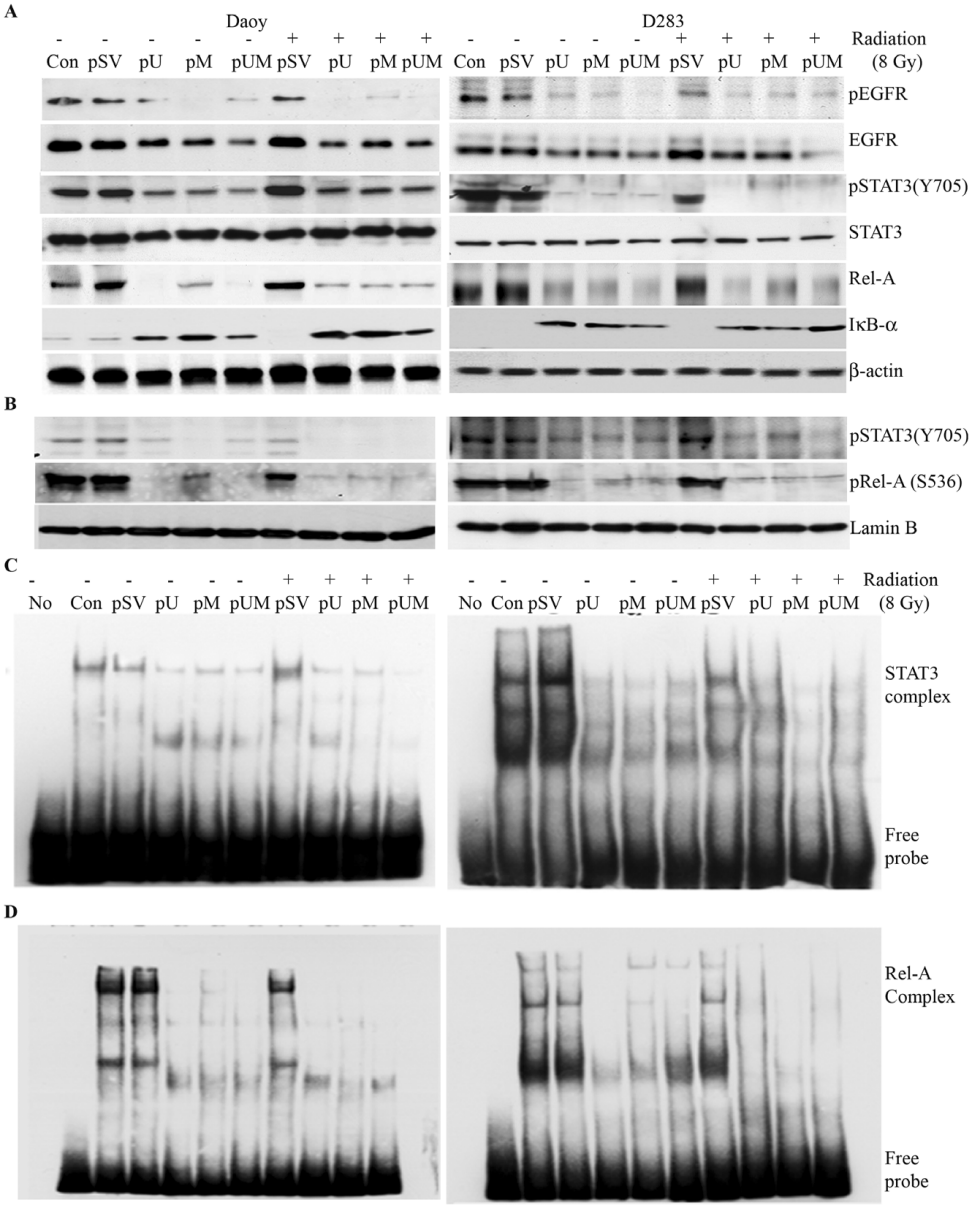
Silencing uPAR and MMP-9 inhibits nuclear levels and activation of STAT3 and NF-κB p65 (Rel-A). A) Total cell lysates were evaluated by immunoblotting to determine the expression of NFκB p65 (Rel-A), IκBα, total and phosphorylated forms of EGFR and STAT3. Uniform loading of the respective protein was confirmed by re-probing the membrane with β-actin antibody. **B)** Nuclear levels of phosphorylated STAT3 and Rel-A were determined by analyzing the nuclear extracts isolated from transfected cells by western blotting. **C–D)** Nuclear extracts were prepared from cells transfected with pU, pM and pUM (with and with radiation), and the DNA binding activity of the nuclear extracts to **C)** STAT3 and **D)** NFκB p65 probe was determined using Electrophorotic Mobility gel Shift Assay. All experiments were repeated three times.

We next assessed the associated signaling pathways activated by uPAR and MMP-9 in regulating the expression of Bcl-2 family members. Both uPAR and MMP-9 directly or indirectly associate with variety of cell receptors and activate intracellular signaling pathways. Our results showed that silencing uPAR and MMP-9 in the medulloblastoma cells significantly inhibited the activation (phosphorylation) of EGFR (Fig. 4A). Knowing the role of EGFR in activating STAT signaling pathway, when examined for the phosphorylated form of STAT3 and STAT5, we noticed a significant inhibition in the phosphorylated forms of STAT3 (Y705) in pUM-transfected cells compared to pSV-transfected cells (Fig. 4A). Given the role of STAT3 as a transcription factor, we initially determined nuclear levels of phosphorylated STAT3 and next compared the binding activity of nuclear extracts to the STAT3 DNA probe between the pSV-treated cells to the pU-, pM-, and pUM-transfected (with and without radiation) cells. STAT3 is constitutively active in control cells and silencing of uPAR and MMP-9 inhibited the nuclear levels of phosphorylated STAT3 (Fig. 4B). In addition, electrophoretic mobility shift assay (EMSA) showed that downregulation of uPAR and MMP-9 (with and without radiation) significantly inhibited binding activity of the respective nuclear extract to the STAT3 DNA probe as compared to either control or pSV-transfected cells (Fig. 4C). Apart from STAT3 we even noticed that the levels of phosphorylated NF-κB p65 (Rel-A; Ser 536) was significantly decreased in pU-, pM- and pUM-transfected cells (with and without radiation) compared to cells treated with pSV or the radiation control (Figs. 4A and 4B). In addition, levels of the NF-κB inhibitor molecule, IκBα, were increased in uPAR and MMP-9-downregulated cells. While our western blot analysis confirmed that downregulation of uPAR and MMP-9 (with or without radiation) inhibited the nuclear levels of phosphorylated Rel-A (Fig. 4B); EMSA results confirmed that nuclear extracts isolates from pU, pM and pUM transfected cells showed a reduced DNA binding activity to NF-κB p65 DNA probe compared to the respective controls (Fig. 4D). We even noticed that apart from Rel-A subunit another subunit of NF-κB complex, p50 also lost the DNA binding activity when uPAR and MMP-9 were down regulated (Fig. S1).

**Figure 5:**
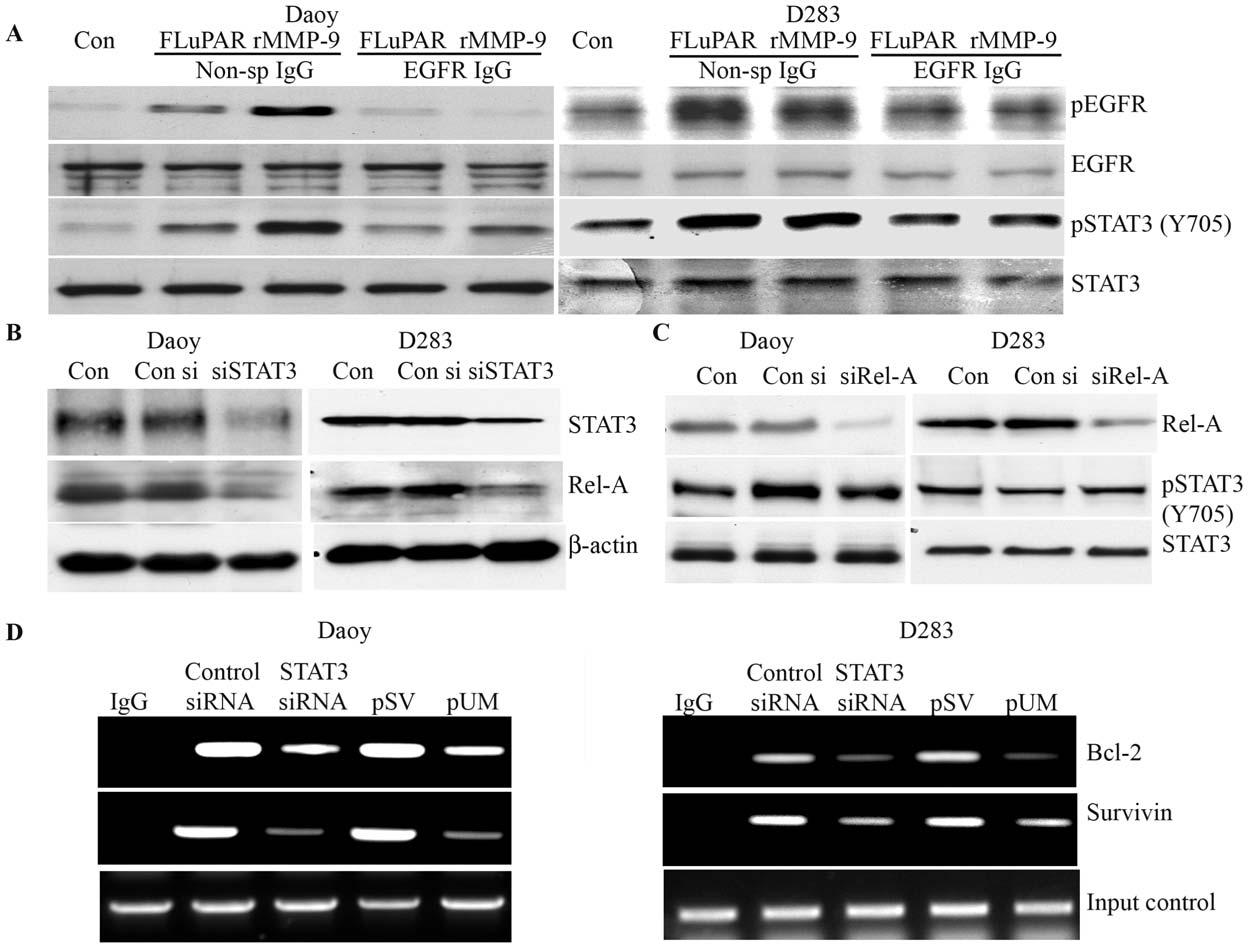
uPAR and MMP-9 activates EGFR and STAT3; Inhibition of STAT3 and NF-κB 65 induces apoptosis in Daoy and D283 cells. To determine possible cross-talk between STAT3 and Rel-A during apoptosis, each gene was downregulated using specific siRNA followed by determination of the nuclear levels of STAT3 and Rel-A in both Daoy and D283 cells. **A)** Medulloblastoma cells transfected with full-length uPAR expressing plasmid or/and incubated with recombinant MMP-9 (25 ηg/ml), were either blocked with EGFR neutralizing antibody or non-specific isotype IgG to determine the role of the extracellular proteases in activation of EGFR and STAT3. Western blot analysis was carried out on cell lysates isolated from Daoy and D283 to determine the levels of phosphorylated EGFR and STAT3. **B)** Western blot analysis was carried out on the nuclear extracts isolated from STAT3-downregulated cells. Membranes were probed with phosphorylated forms of STAT3 and Rel-A. **C)** Similarly, nuclear extracts isolated from Rel-A downregulated cells were evaluated by Western blotting to detect phosphorylated forms of Rel-A and STAT3 in the nucleus. **D)** Chromatin immunoprecipitation assay was carried out with the nuclear extracts isolated from Day and D283 cells transfected with either STAT3 siRNA or pUM plasmid. Chromatin was immunopreciptated with STAT3 and analyzed by PCR using primers specific for Bcl-2 promoter region to determine the STAT3 recruitment at Bcl-2 promoter sequences. Chromatin immunoprecipitated with isotype IgG was used as negative control. Input DNA was confirmed by amplifying the Bcl-2 promoter form the chromatin aliquot collected prior to immunoprecipitation step.

In an independent experiment we attempted to confirm that uPAR and MMP-9 induces the transactivation of EGFR in medulloblastoma cells lines. We observed that either expressing full length uPAR (FLuPAR) or supplementing recombinant MMP-9 activated the phosphorylation of both EGFR and STAT3 (Y705). Our antibody blocking experiments provided further evidence that uPAR/MMP-9 activates EGFR/STAT3 signaling. 24 hrs after transfecting with FLuPAR plasmid or 1 hr prior to supplementing with rMMP-9 (25 ηg/ml), Daoy and D283 cells were incubated either with EGFR IgG’s (50 µg/mL) or Isotype IgG. Blocking EFGR significantly inhibited the uPAR and MMP-9 induced EGFR and STAT3 activation in medulloblastoma cells compared to the cells treated with the isotype IgG (Fig. 5A). Our results confirm that uPAR and MMP-9 regulate EGFR/STAT3 regulated signalling pathway in medulloblastoma.

### STAT3 Inactivation Induces Apoptosis by Transcriptional Regulating the Expression of Bcl-2 and Survivin

We have shown that uPAR and MMP-9 downregulation reduced the activities of STAT3, NF-κB and induced apoptosis. To study the possible link between STAT3 and NF-κB and their role in inducing apoptosis, we downregulated STAT3 and NF-κB (Rel-A) using specific siRNAs to examine apoptotic cell percentage in each of the treatment condition. Knockdown of STAT3 not only downregulated the expression of total and phosphorylated STAT3, but also inhibited the nuclear levels of phosphorylated Rel-A in medulloblastoma cell lines (Fig. 5B). However, we noticed that knockdown of Rel-A specifically downregulated Rel-A with no significant effect on phosphorylated STAT3 (Fig. 5C). Collectively, our results suggested that STAT3 coordinates the activity of NF-κB but not vice versa. Next, using the Apo BrdU TUNEL assay we determined that silencing STAT3 as well as Rel-A in D283 cells in Daoy and D283 cells induced apoptosis. Flow cytometry analysis showed 46% of Daoy and 51% of D283 cells transfected with STAT3 siRNA were TUNEL-positive cells (Fig. S2). Knowing the role of STAT3 as transcriptional factor, the association STAT3 with anti-apoptotic gene regulation was determined. We analyzed the nuclear extracts of pUM and STAT3 knockdown cells by CHIP assay. The chromatin immunoprecipitated with either STAT3 antibodies or isotype IgG’s was subjected to PCR analysis using specific primers amplifying the promoter region of Bcl-2 and survivin genes. PCR analysis of antibody pull downed chromatin showed reduced amplification of Bcl-2 and survivin promoter regions in siSTAT and pUM treated cells compared to control cells. CHIP analysis results confirmed that gene silencing of uPAR/MMP-9 or STAT3 showed reduced recruitment of STAT3 protein at the promoter region of Bcl-2 compared to cell treated with pSV or control siRNA (Fig. 5D).

### Characterization of Tumors from Mice Treated with pU, pM and pUM Alone and in Combination with Radiation

**Figure 6:**
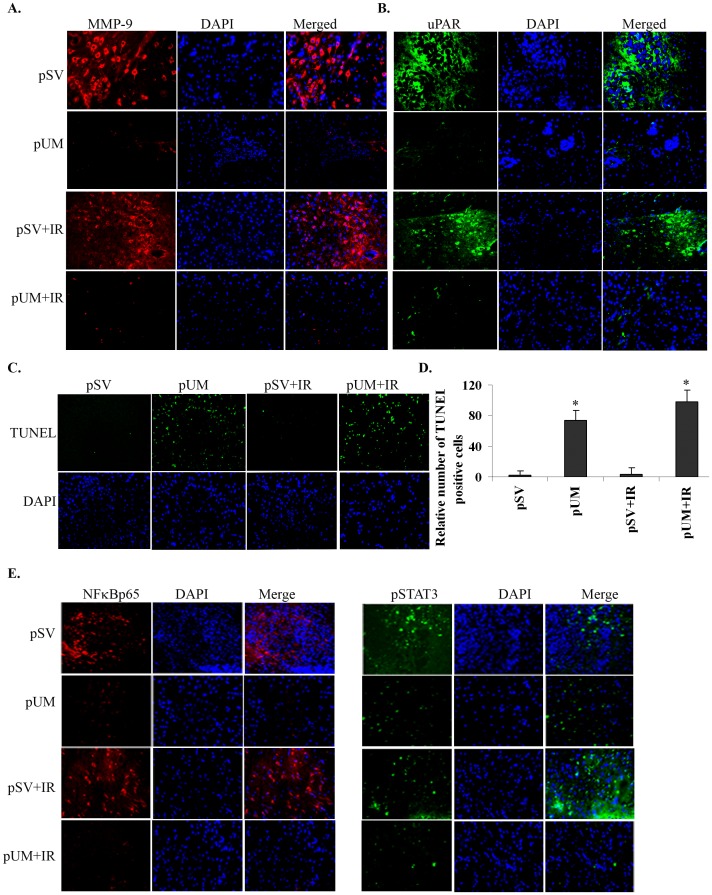
Inhibition of uPAR and MMP-9 induces apoptosis *in vivo*. Daoy cells were implanted intracranially in nude mice, which were treated with control pSV or pUM (150 µg) either alone or in combination with radiation as described in Materials and Methods. **A–B)** Paraffin-embedded brain tumor sections from mice that received control shRNA or pUM either alone and combination of radiation were analyzed by immunohistochmeical analysis using antibodies for **A)** uPAR and **B)** MMP-9 followed by treatment with secondary antibody conjugated with Alexa Fluor 594 and 488 fluorescent tagged. **C)** Induction of apoptosis in pUM-treated tumors was confirmed by carrying out TdT-mediated nick end labeling (TUNEL) assay. The tissues were analyzed for apoptosis using fluorescence microscopy. **D)** The relative number of TUNEL-positive cells was counted and represented graphically. Bars represent the means ± s.d. from three independent experiments, * *p*<0.05. **E)** Immunoflourscence analysis of paraffin-embedded brain sections to detect the levels of NF-κB p65 and Stat3 in pUM and pSV treated mice (with and without radiation). After incubating with the anti Rel-A and Stat3 antibodies for overnight, the tissue sections were washed and incubated in secondary antibody conjugated with Alexa Fluor 594 and 488 fluorescent tagged, respectively. Representative images from three independent experiments were shown in each panel.

We evaluated the effect of uPAR and MMP-9 knockdown, either alone or in combination with radiation treatment, in pre-established tumor growth. Hematoxylin and eosin (H&E) staining of the brain section clearly showed the dense staining in pSV and pSV+IR treated medulloblastoma, representing cancer cells. While the brain section from mice (medulloblastoma) treated with pUM with or without radiation showed relatively sparsely distributed cancerous cells (Fig. S3). Immnuohistochemistry (IHC) analysis of paraffin-embedded tumor sections with uPAR and MMP-9 specific antibodies was performed in control and pUM-treated (with and without radiation) mice. Consistent with our *in vitro* results, tumor sections from mice treated with the shRNA constructs showed decreased staining for uPAR and MMP-9 as compared to pSV-treated tumors (Figs. 6A and 6B). Next, the induction of apoptosis in the tumors treated with pUM was determined by carrying out TUNEL assay of the brain tumor sections. The results confirmed that pUM treatment (with and without radiation) significantly increased DNA fragmentation of the tumor cells compared to tumors treated with pSV (Fig. 6C). Treatment with pUM alone resulted in more than 65% of cells being TUNEL-positive as compared to pSV-treated tumors (Fig. 6D). Notably, the combination of pUM with IR resulted in nearly 80% of cells being TUNEL-positive cells as compared to the pSV and IR-treated tumors. Next, we attempted to determine the levels of NF-κB p65 and STAT3 in tumors treated with pSV and pUM (with and without radiation). IHC analysis revealed that the expression of NF-κB p65 (Rel-A) and STAT3 molecules were significantly decreased in the brain section of pUM treated mice (with and without radiation) compared to brain section of pSV treated mice (Fig. 6E). Similarly, we even noticed a significant decrease in EGFR staining in brain section of pUM treatment mice compared to pSV treated mice. However, intensity of Bak staining in pUM-treated tumor sections was higher when compared to pSV-treated tumor sections (Fig. S4).

## Discussion

Earlier we reported irradiation treatment enhanced tumor growth and metastasis [21], [22] and transfecting medulloblastoma cells with pU, pM and pUM either alone or in combination with radiation successfully regressed cell proliferation [23]. In the present study, molecular mechanisms associated with downregulation of uPAR and MMP-9 in the induction of apoptosis was explored. Several reports mentioned that blocking the activities of uPAR and MMP-9 resulted in apoptosis in various cancer cells [21], [24]–[26]. Growth in number of studies implicating multiple roles of uPAR and MMP-9 in regulating extracellular matrix dissolution, activating growth factor, initiating intracellular signaling leading to tumor progression and metastasis are increasing day by day [8]. Knocking down the expression of uPAR and MMP-9 significantly inhibited uPAR and MMP-9 levels and altered downstream signaling molecules, thereby leading to transcriptional inhibition of anti-apoptotic molecules and directing the cells towards apoptosis. Further we have established that combining radiation treatment to the pU, pM and pUM transfected medulloblastoma cells showed a higher efficiency in inducing apoptosis.

Apoptosis is mainly activated by either extrinsic (ligand/receptor mediated) or intrinsic (mitochondrial) signaling pathways [27] **.** Induction of mitochondrial apoptosis requires involvement of the Bcl-2 family, including anti-apoptotic gene products (e.g., Bcl-2, Bcl-xL) and pro-apoptotic gene products (e.g., Bax, Bak, Bcl-xS, Bim) [28], [29]. Therefore, by assessing the mitochondria-derived factors mediating the cell death process we successfully demonstrated that inhibition of uPAR and MMP-9 decreased the expression of Bcl-2 and Bcl-xL, activated Bid cleavage, and enhanced Bak expression in medulloblastoma cells. Stepwise activation of pro-apoptotic molecules, such as Bax and Bak, requires either cleaved BID or Bim to initiate mitochondrial apoptosis [30]. Previous studies have shown that blocking uPAR in various cancer cells reduced the expression of Bcl-2 and enhances the expression of Bax [25], [31]. In contrary, we observed that downregulation of uPAR and MMP-9 resulted in elevated expression of Bak with no significant difference in Bax expression. Pro-apoptotic activity of Bak was reported to be distinctly controlled by anti-apoptotic Bcl-2 family members, such as Bcl-xL and Mcl-1 [32]. Results from our studies suggest that apoptotic stimuli generated by downregulation of uPAR and MMP-9 counteract the function of anti-apoptotic Bcl-2 and Bcl-xL molecules and activate pro-apoptotic Bak in medulloblastoma cells. Another hallmark of mitochondria-mediated apoptosis is the release of cytochorme C into the cytosol from the mitochondria, which leads to the activation of caspases. Intracellular cleavage of BID and upregulation of Bak in pUM-transfected cells suggests association of mitochondria membrane collapse during the induction of apoptosis [33] **.** Further, mitochondrial apoptosis was reported to be mediated by membrane potential damage and release of cytochrome C from mitochondria to the cytosol [34]. Taken together, results of the present study confirmed that inhibiting uPAR and MMP-9 effectively increased the Bak/Bcl-xL ratio, which altered the membrane potential of mitochondria, triggered the translocation of cytochrome C into the cytosol and subsequently leading to activate the effectors of apoptosis, caspases.

Being extracellular proteases, both uPAR and MMP-9 coordinates with other surface receptor to activate intracellular signaling regulating cancer cell progression, invasion, migration and angiogenesis. Among several cell surface receptors, the association of uPAR with epidermal growth factor receptor (EGFR) is well studied [35]. Even in the present study, we determined that the knockdown of uPAR and MMP-9 reduced the expression of total EGFR in medulloblastoma. EGFR is known to initiate several intracellular signaling pathways such as Ras-MAPK, PI3K-Akt, and STATs [36]–[38]. Further we established that expressing uPAR or by supplementing rMMP-9 in the culture media activate EGFR. Subsequently our antibody blocking experiment confirmed that blocking EGFR inhibited the activation of uPAR/MMP-9 induced STAT3 in medulloblastoma cell lines. With the growing functional importance of STAT3 as a transcription factor that modulates cell proliferation and apoptosis [39], we determined the levels of activated STAT3 in medulloblastoma cells transfected with pU, pM and pUM (with and without radiation). The significant reduction of phosphorylated STAT3 in uPAR and MMP-9-downregulated cells confirmed that EGFR transactivation might be involved in activation of STAT3. The data collectively confirmed that downregulation of uPAR and MMP-9 reduced the transactivation of EGFR, which in turn might potentially inhibit the activation of STAT3. Ealrier reports also mentioned that downregulation of STAT3 induced apoptosis in human glioma [40] and breast cancer cells [41] **.** Taken together, the data from the present study suggest that apoptosis induced in uPAR and MMP-9-downregulated medulloblastoma cells might be due to inactivation of the STAT3-related signaling pathway. Moreover, it was reported that EGFR-mediated activated STAT3 translocates into the nucleus and regulates the transcription of genes associated with cell survival [42]. We showed that nuclear levels and the transcriptional DNA binding activity of STAT3 in uPAR and MMP-9-downregulated cells were significantly reduced. Participation of STAT3 in oncogenesis was reported by up regulation of genes encoding apoptosis inhibitors (Mcl-1, Bcl-2, survivin etc.). Our further investigation showed that downregulation of uPAR and MMP-9 inhibited the transcriptional activity of STAT3 in regulating the expression of Bcl-2 and survivin in medulloblastoma.

NF-κB most commonly antagonizes apoptosis by activating the expression of anti-apoptotic proteins and antioxidant molecules [43]. In the present study, we demonstrate that nuclear levels of the phosphorylated Rel-A (the p65 subunit of the NF-κB complex) and NF-κB DNA binding activity was significantly inhibited in uPAR and MMP-9-silenced medulloblastoma cells. Regulation of apoptotic behavior by NF-κB either in a pro- or anti-apoptotic manner is determined by the nature of apoptotic stimuli. Moreover STAT3 was reported to acts as target for inducing apoptosis in solid and hematological tumors [44]. Persistently activated STAT3 was reported to be required for maintaining the constitutive NF-κB activity in melanoma and prostate cancer cells [45]. Possible molecular cross-talk between STAT3 and NF-κB signaling was reported previously in various cancer cells [46]. Similarly our studies showed that by down regulating STAT3 levels in medulloblastoma cells lines, the levels of Rel-A were also reduced. Further, in our studies we noticed a significant down regulation of inhibitory apoptotic molecules such as Survivin, XIAP and cIAPI which might have resulted due to the inhibition of STAT3/NF-κB activity. Several reports showing that inhibition of STAT3 activity results in down regulation of Survivin following irradiation [47], [48].

In sum, the present study demonstrated that siRNA-mediated downregulation of uPAR and MMP-9 significantly inhibited the STAT3 activity which regulated the transcription of inhibitory apoptotic molecule(s). Our results also suggest that NF-κB acts as a downstream effector of STAT3 and persistently activated STAT3 is required for maintaining the constitutive NFκB activity in Daoy and D283 cells. In conclusion, we demonstrate that downregulation of uPAR and MMP-9 inactivates STAT3/NF-κB signaling to induce apoptosis under both *in vitro* and *in vivo* conditions.

## Materials and Methods

### Cells Lines, Transfections and Radiation

Human medulloblastoma cancer cell lines, Daoy and D283, were obtained from American Type Culture Collection (Manassas, VA) and grown in advanced MEM and Improved MEM media, respectively, containing 10% fetal bovine serum and 1% penicillin/streptomycin. All cell lines were maintained in a 37°C incubator in a 5% CO_2_ humidified atmosphere. Monocistronic plasmids expressing shRNA targeted against uPAR (pU), MMP-9 (pM) individually, a bicistronic construct targeting both genes simultaneously (pUM) and a vector expressing a scrambled non-specific human sequence (pSV) were constructed as described previously [49]. Plasmid expressing full length uPAR gene was purchased from Origene (Rockville, MD), and recombinant MMP-9 was purchased from R&D systems (R&D Systems, Minneapolis, MN). Cells were transiently transfected with either the plasmid constructs using Fugene HD (Roche Applied Science, Madison, WI) as per the manufacturer’s instructions. Briefly, cells (70% confluent) were transfected with plasmids and incubated for 48 to 72 hrs before collected the cells for further analysis. Cell treated with transfection reagent alone was used as control (mock). As a radiation source we used RS 2000 Biological Irradiator (Rad Source Technologies, Inc., Boca Raton, FL). In case of combining the radiation treatment with shRNA treatment, 48 hrs after transfecting with shRNA plasmids or mock the cells were exposed to ionizing radiation (8 Gy) and incubated for another 16 to 24 hrs before collecting the cells for further analysis.

### Semi-quantitative Reverse Transcription PCR

Two µg of the total RNA extracted from cells using TRIZOL reagent was used for the synthesis of first strand cDNA using Transcriptor First Strand cDNA Synthesis Kit (Roche Applied Science, Indianapolis, IN). PCR reaction was performed to amplify the cDNA of the mRNA transcript using the following primers: MMP-9∶5′-TGGACGATGCCTGCAACGTG-3′ (forward) and 5′-GTCGTGCGTGTCCAAAGGCA-3′ (reverse); uPAR: 5′-TTACCTCGAATG CATTTCCT-3′ (forward) and 5′- TTGCACAGCCTCTTACCATA -3′ (reverse).

### Chromatin Immunoprecipitation (CHIP) Assays

CHIP assay was performed using ChIP-IT™ Express Magnetic Chromatin Immunoprecipitation kit following manufacturer’s protocol (Active motif, Carlsbad, CA). After the respective treatment, chromatin isolated from the formaldehyde fixed cells was allowed to shear by sonication. The chromatin DNA was immunoprecipitated with STAT3 and Normal mouse IgGs by incubation at 4°C for 2–4 hours on a rotor. Nearly 10 ηg of immunoprecipitated DNA was used to detect STAT3 binding to Bcl-2 and survivin promoter sequences by standard PCR analysis. The amplicons were resolved on agarose gels and visualized under UV light. Bcl-2 primers used in the present study are sense 5′ – GTTGGTCAGCAGTTCCAAA-3′ and antisense 5′-ATTTTTCCCCACACACCAAG-3′; while survivin primers used are sense 5′- AAACGAGG GCAATGTGAATC -3′ and antisense 5′- CACAGCAATTCTCTGCCTCA -3′.

### APO-BrdU TUNEL Assay

Cells were harvested 72 hrs after Transfection, fixed and the apoptotic cells were detected using the APO-BrdU TUNEL Assay Kit (Molecular Probes Inc, Eugene, OR) as per the manufacturer's protocol. Controls were established with the positive and negative cells provided in the kit using the same protocol.

### Annexin V/PE Staining Assay

Transfected cells were harvested, washed and re-suspended with PBS. Apoptotic cells were identified by double supra-vital staining with Annexin V and PI, using the Annexin V/PE Apoptosis Detection kit (BioVision, CA) according to the manufacturer's instructions. Flow cytometric analysis and the data acquisition and analysis were performed using a Becton Dickinson flow cytometer with Cell Quest software.

### Measurement of Mitochondrial Membrane Potential (Mitotracker Assay)

The integrity of the mitochondrial membrane (Ψm) was evaluated using MitoLight Mitochondrial Permeability assay kit (Millipore, Danvers, MA). 48 hrs after transfection, the cells were incubated for another 16 hrs after treating either with or without radiation. Cells were trypsinized and incubated at 37°C for 30 minutes in 1 mL complete medium containing 2 µM Mitotracker red (a lipophilic cationic dye). The cells were washed and the number of cells emitting green fluorescence (FL-1 channel) versus cell emitting red fluorescence (FL-2 channel) was recorded by flow cytometry (BD Biosciences, San Jose, CA) and quantified.

### Caspsase Activity Assay

Caspase-3 and caspase-9 activities were determined in the transfected cells using caspase-3 and capsase-9 colorimetric assay kits, respectively, according to the manufacturer’s instructions (Chemicon International Inc., Temecula, CA).

### Immunoblotting

After transfection with pSV, pU, pM and pUM (with and without radiation), cells were lysed (20 mM Tris, 150 mM NaCl, 1 mM EDTA, 1% Triton X-100, 1 mM phenylmethylsulfonyl fluoride and leupeptin). Equal amounts of protein were separated on SDS–PAGE gels, followed by transfer onto nitrocellulose membranes. The blots were incubated with primary antibodies followed by HRP-conjugated secondary antibodies. Immunoblots were developed using the enhanced chemiluminescence (ECL) detection system (Amersham Pharmacia, Piscataway, NJ) according to the manufacturer’s protocol. Antibodies used in the present study: Bak, Bcl-2, Bcl-xL, Bid, Caspase-3, Caspase-9, cIAPI, CyctochromeC, EGFR, phospho EGFR, MMP-9, NF-κB p65, phoshpho NF-κBp65, NF-κBp50, STAT3, phosphoSTAT3, STAT5, Survivin, uPAR, XIAP, GAPDH, Lamin-B, COX IV and α-tubulin were purchased from Santa Cruz biotechnology (SantaCruz, CA). PARP-1 antibody was purchased from EMD Biosciences (San Jose, CA, USA).

### Isolation of Proteins from Mitochondrias

Mitochondrial proteins were isolated using mitochondrial isolation kit (Sigma, St. Louis, MO) as per the manufacturer’s instructions. This pellet was re-suspended in CellLytic M lysis buffer and used for further analysis of mitochondrial proteins.

### Electrophoretic Mobility Shift Assay

Nuclear extracts were prepared from cells 72 hrs after transfection using a nuclear extraction kit (Panomics, Fremont, CA). The interaction of nuclear proteins with the labeled DNA probe was determined by electrophoretic mobility shift assay (EMSA) as per the manufacturer’s instructions. The bands were visualized after exposure to a Hyperfilm-MP autoradiography film (Amersham, Piscataway, NJ).

### Animal Studies and Immunohistochemical Analysis (IHC)

For the intracranial tumor model, Daoy (1×10^6^) cells were intracerebrally injected as described previously [22]. Ten days after tumor implantation, the mice were injected with shRNA (150 µg) towards the area of the brain were the tumor cells where implanted using Alzet osmotic pumps (model 2001, Alzet Osmotic Pumps, Cupertino, CA). Four weeks after tumor inoculation, five mice from each group were sacrificed by cardiac perfusion with 4% formaldehyde in PBS, their brains were removed, and paraffin sections were prepared. Sections were stained with H&E to visualize tumor cells and to examine tumor volume. IHC analysis of the paraffin-embedded brain sections from pSV- and pUM-treated mice (with or without radiation) using specific antibodies.

### Statistical Analyses

Quantitative data from cell counts, Western blot analysis, and other assays were evaluated for statistical significance using one-way analysis of variance (ANOVA). Data for each treatment group were represented as mean ± SEM and compared with other groups for significance by one-way ANOVA followed by Bonferroni's post hoc test (multiple comparison tests) using Graph Pad Prism version 3.02, a statistical software package. Results were considered statistically significant at a *p* value of less than 0.01 and 0.05.

### Ethics Statement

The Institutional Animal Care and Use Committee of the University of Illinois College of Medicine at Peoria (Peoria, IL) approved all surgical interventions and post-operative care.

## Supporting Information

Figure S1 **Nuclear extracts isolated from Daoy and D283 cells transfected with pSV, pU, pM and pUM (with or without radiation treatment) were probed with NFκBp50 DNA probe to determine the DNA binding activity using Electrophorotic Mobility gel Shift Assay.**
(TIF)

Figure S2 **Daoy and D283 cells transfected with siRNA either against STAT3 or siRel-A were analyzed by Apo BrdU TUNEL assay.** TUNEL-positive apoptotic cells were analyzed by flow cytometry. Alexa Fluor 488 fluorescent-tagged IgG was used to detect Apo-BrdU antibody.
(TIF)

Figure S3 **H&E (Hematoxylin and eosin) stained brain section of mice medulloblastoma treated with either pSV or pUM, with or without radiation.**
(TIF)

Figure S4 **The levels of EGFR and Bak in the paraffin-embedded brain sections of pUM and pSV treated mice were determined by immunohistochemistry using specific antibodies followed by incubation with HRP-conjugated secondary IgG.** The complex was further detected by DAB staining. Representative images of each treatment are shown.
(TIF)
