# Expression of Concern: Apoptosis Induced by Knockdown of uPAR and MMP-9 is Mediated by Inactivation of EGFR/STAT3 Signaling in Medulloblastoma

**DOI:** 10.1371/journal.pone.0328377

**Published:** 2025-07-15

**Authors:** The PLOS One Editors

After the publication of this article [1], concerns were raised about the results presented in Figs 2-4. Specifically,

- The Fig 2A Daoy GAPDH panel and the Fig 4B D283 Lamin B panel appear similar
- The Fig 2B D283 Cox IV panel and the Fig 3C Daoy GAPDH panel appear similar

In addition to the above concerns, the Fig 5A Daoy STAT 3 panel in this article [1] appears similar to the Fig 3C β-actin panel in [2, retracted in 3] and the Fig 6 AKT panel in [4]. PLOS note that [2, retracted in 3] and [4] were published later by researchers that do not appear to share affiliations with the authors of [1].

The authors did not respond to the journal’s communications.

The *PLOS One* Editors issue this Expression of Concern to alert readers of the panel overlap concerns affecting Figs 2-4, which call into question the reliability and validity of the published results.
