# Supplementary figures and images for: Apoptosis Induced by Knockdown of uPAR and MMP-9 is Mediated by Inactivation of EGFR/STAT3 Signaling in Medulloblastoma
Source: PLoS One. 2012 Sep 12;7(9):e44798. doi: 10.1371/journal.pone.0044798 (PMC3440337; doi:10.1371/journal.pone.0044798)

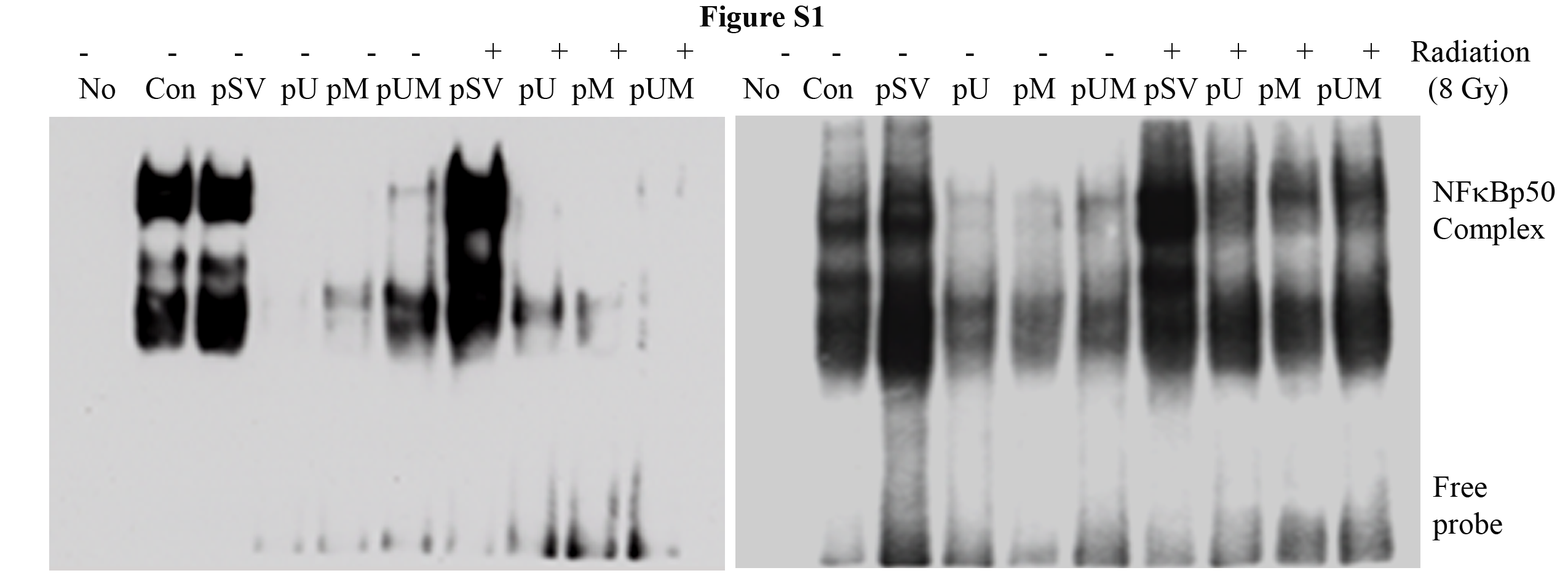

Supplement: Figure S1 — Nuclear extracts isolated from Daoy and D283 cells transfected with pSV, pU, pM and pUM (with or without radiation treatment) were probed with NFκBp50 DNA probe to determine the DNA binding activity using Electrophorotic Mobility gel Shift Assay. (TIF) [file pone.0044798.s001.tif]

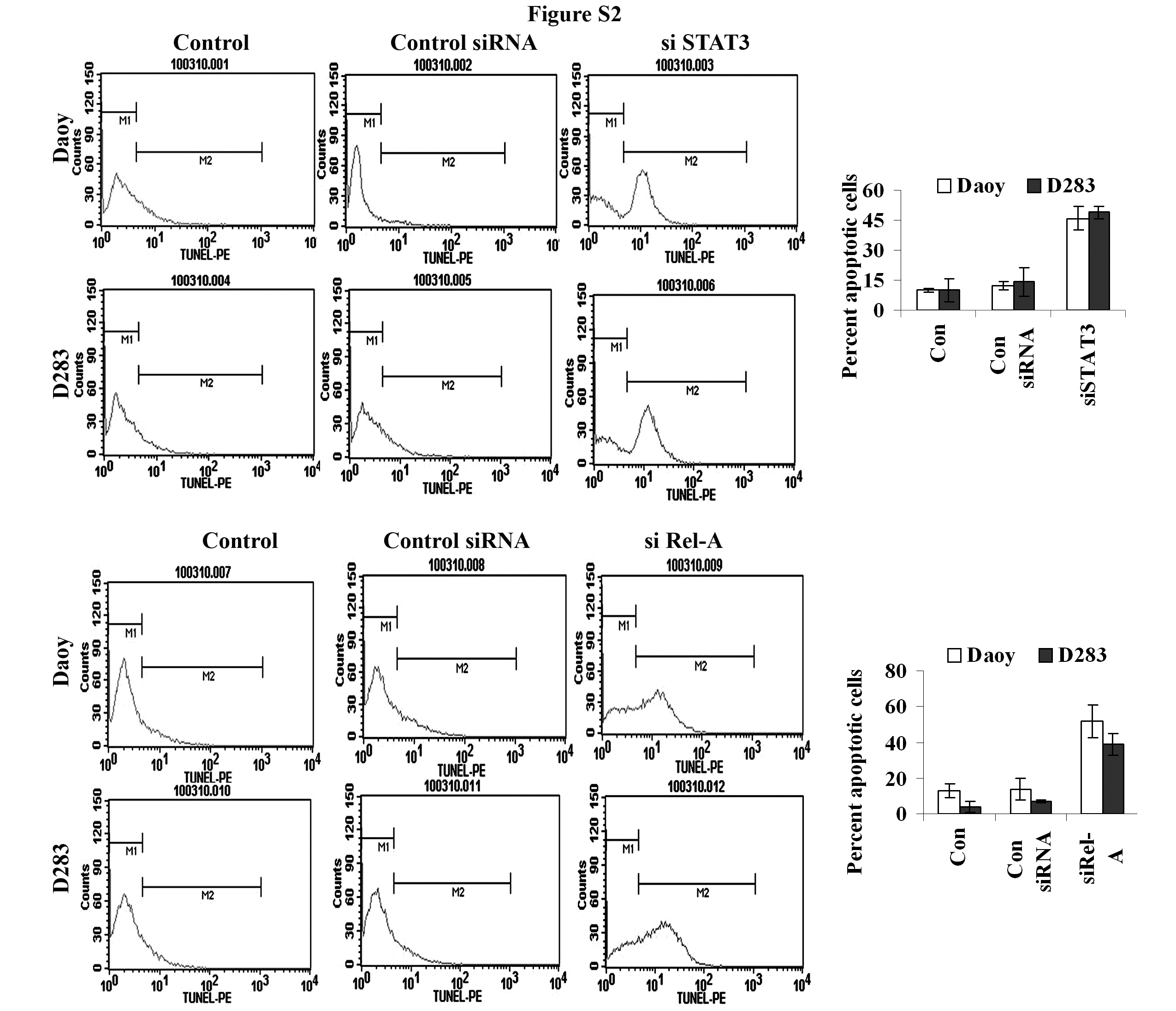

Supplement: Figure S2 — Daoy and D283 cells transfected with siRNA either against STAT3 or siRel-A were analyzed by Apo BrdU TUNEL assay. TUNEL-positive apoptotic cells were analyzed by flow cytometry. Alexa Fluor 488 fluorescent-tagged IgG was used to detect Apo-BrdU antibody. (TIF) [file pone.0044798.s002.tif]

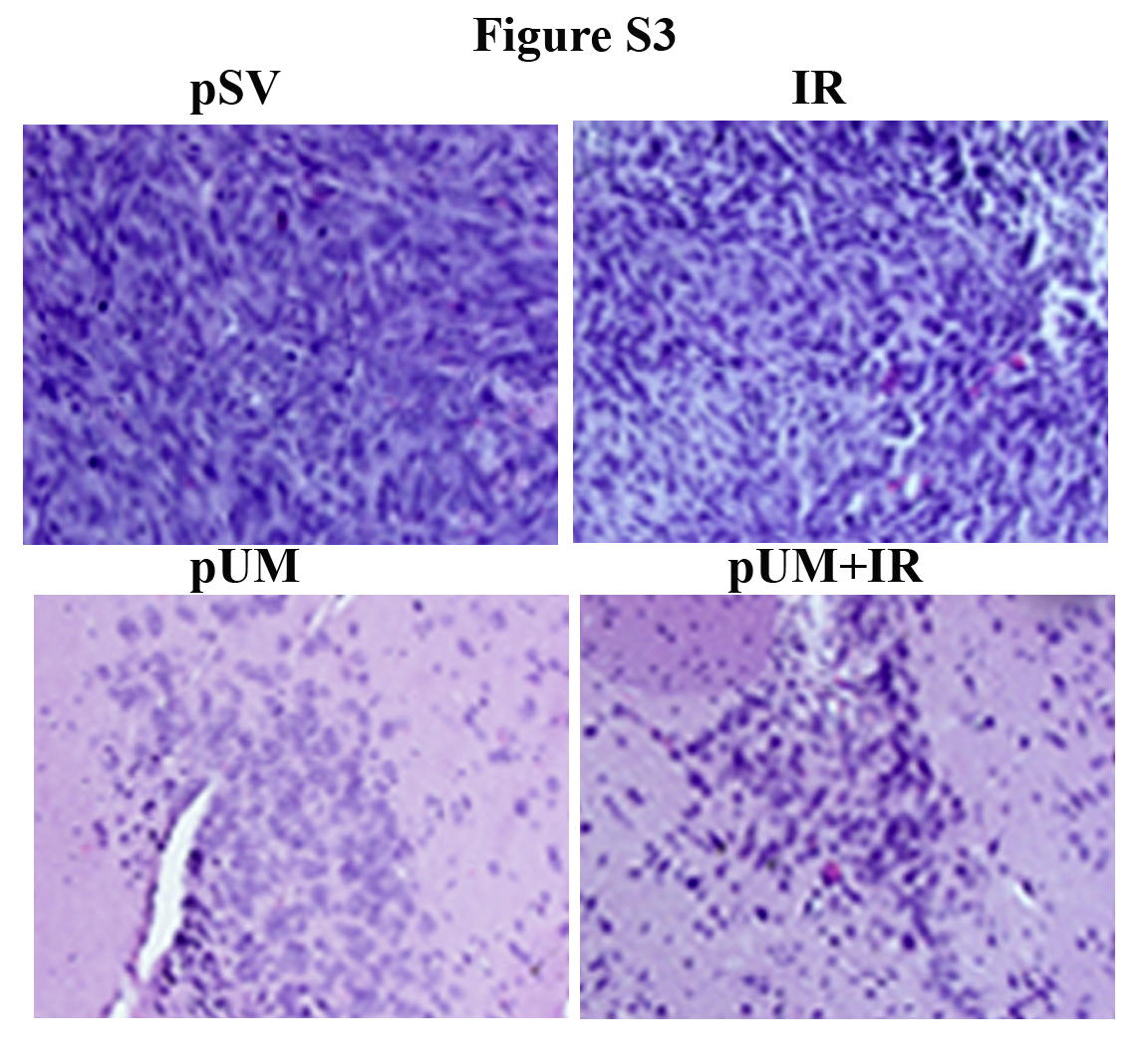

Supplement: Figure S3 — H&E (Hematoxylin and eosin) stained brain section of mice medulloblastoma treated with either pSV or pUM, with or without radiation. (TIF) [file pone.0044798.s003.tif]

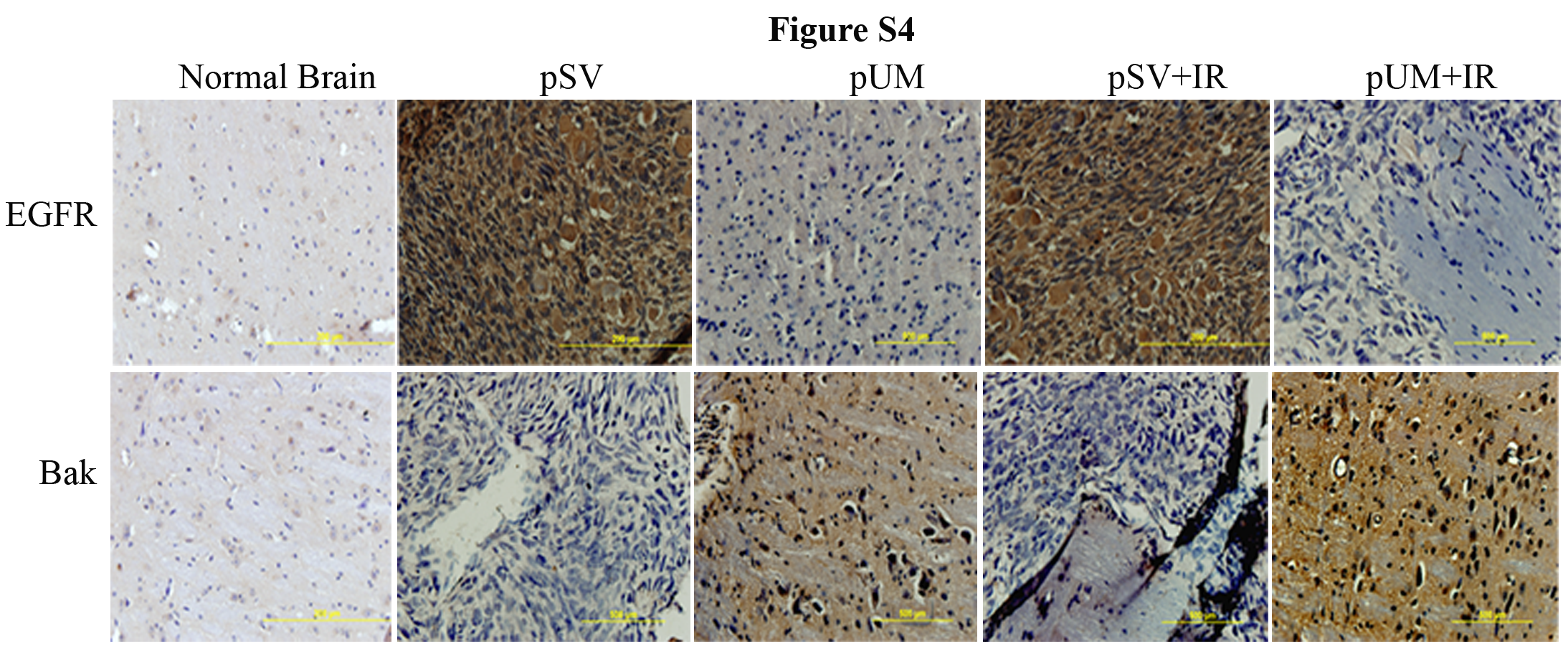

Supplement: Figure S4 — The levels of EGFR and Bak in the paraffin-embedded brain sections of pUM and pSV treated mice were determined by immunohistochemistry using specific antibodies followed by incubation with HRP-conjugated secondary IgG. The complex was further detected by DAB staining. Representative images of each treatment are shown. (TIF) [file pone.0044798.s004.tif]
